# The role of pre-school teachers in the control of soil-transmitted helminthes in coastal region, Kenya

**DOI:** 10.1186/s40794-016-0040-y

**Published:** 2016-10-13

**Authors:** D. W. Njomo, J. Masaku, G. Odhiambo, R. Musuva, F. Mwende, E. Matey, I. G. Thuita, J. H. Kihara

**Affiliations:** 1 Eastern and Southern Africa Centre of International Parasite Control, KEMRI, P.O. Box 54840-00200, Nairobi, Kenya; 2 Centre for Global Health Research, KEMRI, P.O. Box 1578-40100, Kisumu, Kenya; 3 Centre for Microbiology Research, Kenya Medical Research Institute, KEMRI, P.O. Box 54840-00200, Nairobi, Kenya; 4 Ministry of Education, Science and Technology, P.O. Box 30040-00100, Nairobi, Kenya; 5 Ministry of Health, Division of Vector Borne Diseases, P.O. Box 30016-00100, Nairobi, Kenya

**Keywords:** Pre-school age, Children, Deworming, Soil-transmitted helminthes

## Abstract

**Background:**

Soil transmitted helminthes (STH) are significant health problems among school-age children. In Kenya’s coastal region, the prevalence among pre-school age children (PSAC) ranges from 27.8 to 66.7 %. Whereas some pre-schools are as far as 7 km from the nearest primary schools, the National School-Based Deworming Programme (NSBDP) requires the pre-school teachers to walk with the children to primary schools for deworming by trained primary school teachers. The long distances may contribute in making drug delivery ineffective and unsustainable.

**Methods:**

To assess the pre-school teachers’ knowledge, experiences and perceptions of STH and the NSBDP, a cross-sectional study using qualitative methods was conducted in four sub-counties of the Coast Region. Through purposive sampling, 41 pre-schools which are 2 or more kilometers away from a primary school were selected and in-depth interviews administered to the teachers. Separate in-depth interviews were administered to 34 community health extension workers, 40 opinion leaders and 38 primary school teachers all purposively selected to assess their perceptions of the role of pre-school teachers in the NSBDP. Data was audio recorded, transcribed, coded and analyzed manually by study themes.

**Results:**

A third of the pre-school teachers were aware of signs of STHs and a half indicated that poor hygiene and sanitation practices are major causes. A majority of the pre-school teachers reported that health education and environmental sanitation are key control methods. Majority (39) had received information on NSBDP from various sources and all took part in community sensitization and in treating the pre-school children. A large majority of all study participants indicated that treating the children at pre-schools is ideal for increased coverage. Majority of the pre-school teachers perceived the NSBDP as important in improving the health status of the children. All study participants felt that the parents needed to be given adequate information on STHs and training the pre-school teachers to assist in community sensitization and drug administration would be useful.

**Conclusion:**

Pre-school teachers are a potential resource to the NSBDP that should be utilized to instill proper water and sanitation practices to the young children and assist in community sensitization. They should be empowered and allowed to administer treatment for STH control. County Governments, their current employers should find ways of engaging them in worm control efforts.

**Trial registration:**

KEMRI SSC 2547, Registered 22 July 2013.

## Background

Neglected tropical diseases (NTDs), a group of tropical diseases affecting more than one billion poor people worldwide living at the periphery of health systems [1] can cause disability, disfigurement, under nutrition and cognitive impairment. Soil-transmitted helminthes (STHs) spp *Ascaris lumbricoides*, *Trichuris trichiura* and hookworm, contribute the greatest disease burden among the NTDs [2]. Pre-school age children are among the priority groups identified by the World Health Organization that harbor chronic and intense infection [3] thus having compromised nutritional status and cognitive abilities [4]. In Kenya, the endemicity of STHs among pre-school age children is high with sub-counties in Coast region having prevalence ranging from 27.8 to 66.7 % and current estimates suggest that up to 3 million school-age children (SAC) live in areas that warrant mass treatment for STH infection [5]. A high percentage of infected children means that the environment becomes more heavily contaminated which in turn increases the risk of infection for the whole community. Responding to the need of the 2010 target of treating 75 % of school-age children, a new consortium was established in Paris in 2014, to support countries in coming up with ways of addressing STH among preschool-age (PSAC) and SAC [6].

In helminthiasis-endemic countries which are usually developing countries the education sector has for a long time been used for delivering of medicines for parasite control because the school network is usually better established than the health system [7]. Regular school-based deworming is a proven, cost-effective strategy that can avert the health and educational consequences of STH infections [8] and partners are already helping support national deworming programmes, either as part of comprehensive school health programs or integrated programs tackling neglected tropical diseases (NTDs). Preschool-age children, school-age children, and women of child-bearing age are the segments of people at most risk of STH morbidities [9, 10]. However, programmes have traditionally excluded children between the age of 2 and 6 years who attend pre-schools. Indeed, in many low-income countries, health services for children above 2 years of age are more limited than the Maternal Child Health (MCH) services provided to younger children. One public sector solution is to expand access to MCH “health days” to include children up to 6 years as has been tried in Uganda [11]. Even in developing countries, pre-school age programmes during the early vulnerable years are usually outside the public sector and are commonly implemented by civil society organizations and the private sector [12]. This age bracket requires to be treated as they also carry a heavy worm burden and pose a risk of re-infecting the treated SAC while interacting and playing at the community level. Reducing the number of worms in all age groups of children is beneficial to everyone [13].

In Kenya, hookworm occurs throughout most parts of the country, with areas of highest prevalence being found in south-western Kenya and in Kilifi and Kwale counties on the Coast Region. *Ascaris lumbricoides* and *Trichuris trichiura* are most prevalent in western Kenya and in the coastal areas, especially Lamu and Tana River counties [5]. The NSBDP in Kenya was launched in 2009 (with albendazole) and 2012 with praziquantel administered in prioritized areas, and by 2013 over 6 million SAC had been treated [14]. The results of a survey conducted prior to deworming in 2012 showed that the prevalence of STH in pre-schools in Matuga and Msambweni sub-counties in Kwale County was 27.8 and 66.7 % respectively while that of Malindi sub-county in Kilifi County was 44.5 % [15]. Annual mass drug administration using trained primary school teachers has been going on targeting all pre-school and school age children as recommended by World Health Organization [16]. The results presented in this paper are part of a larger study entitled “Evaluating Different Drug Delivery Approaches for Treatment of STHs among Pre-School Age Children during the National School-Based Deworming Programme of Kenya” which was conducted after the Mass Drug Administration of 2014. The current study sought to assess the pre-school teachers’ knowledge of STH signs, causes, transmission and prevention and their awareness, perceptions of and experiences during NSBDP with a view of understanding the role that they can play to improve the Programme implementation.

## Methods

### Study site

The study was conducted in four STHs endemic sub-counties (Matuga, Msambweni, Lunga Lunga and Malindi) in coastal Kenya [15]. Three of the sub-counties are located in Kwale County while Malindi sub-county is located in Kilifi County. Kwale County is located, 40 km south of Mombasa, the second largest city in Kenya, which has an area of 8360 km2 with a population of 649,931 persons [17] and lies at an altitude of between 60 and 135 m above sea level. The County has 3 hospitals, 5 health centers, 37 government dispensaries, and 3 private dispensaries. Accessibility of health services is however low. A majority of the population live over 5 km away from the nearest health facility. Shortage of drugs and lack of diagnostic facilities adversely affect provision of quality health care. Cost of health care system is also a barrier to access to services. The doctor/patient ratio stands at 1:82,690 which in itself is telling of services offered due to shortage of staff in the health facilities. The most prevalent diseases are; malaria, respiratory diseases, diarrhea, intestinal worms, stomachache and flu. The utilization of health facility for child delivery stands at 32 % [18], the main reasons for low levels of use being distances to the nearest health facilities and low socio-economic status.

Malindi sub-county in Kilifi County is located 120 km northeast of Mombasa, and lies between latitudes 2.2 and 4° south and between longitudes 39 and 41° east. It covers a geographical area of 7605 km^2^ with a total population of 384,643 [17]. The sub-county has 3 hospitals (1government and 2 private); 24 dispensaries (17 governments and 7 Non-Governmental Organizations) and 4 private chemists. The average distance to the nearest health facility for urban areas is 1 km and 3 km for rural areas. Most of the health facilities are therefore not accessible to the majority of the population. High poverty levels, cost sharing and long distances inhibit people from visiting these facilities. The doctor/patient ratio is 1:19,502. The most prevalent diseases are; malaria, respiratory diseases, diarrhea, intestinal worms, sexually transmitted infections, anemia and eye infections. The utilization of health facility for child delivery is at 41 % and reasons for low usage are distance and low socio-economic status [19].

### Study design and setting

This was a retrospective cross-sectional study that utilized qualitative methods for data collection. The data was collected in May 2014, after the February 2014 round of deworming. Based on the fact that pre-schools are generally situated far away from primary schools unlike those in Western and Central regions and also due to high prevalence of worm infestation, the 4 sub-counties were purposively selected for the study. A list of the total number of pre-schools and their enrolment was acquired from the Sub-county Early Childhood Development Education (ECDE) Offices of each of the four sub-counties. All pre-schools that are stand alone and are ≥ 2 km away from a primary school were identified with the help of ECDE Office and considered for the study. Fifty percent of the pre-schools in each sub-county were assigned for treatment by the primary school teachers of the nearest primary schools as prescribed by the NSBDP while the remaining half was assigned for treatment by the Community Health Extension Workers (CHEWS) who were tasked with delivering the medication and treating the pre-school children at their learning Centres.

### Study population

The study participants included: community health extension workers (34), pre-school teachers (41), primary school teachers (38) and opinion leaders; social, community and religious groups leaders (40) serving the four sub-counties who were purposively selected for the study. In-depth interviews (IDIs) were administered to the pre-school teachers so as to gather information on their knowledge of STHs, causes, transmission and prevention and their perceptions and experiences during treatment of the pre-school age children in the NSBDP. Separate in-depth interview were administered to the community health extension workers, the primary school teachers and opinion leaders so as to elicit more information on NSBDP and treatment of the pre-school age children. All the data were collected by trained KEMRI personnel. Notes were taken during the IDIs and audiocassettes used to tape record all the information using *Kiswahil*i, a commonly used language in the study area. Standard procedures used when conducting IDIs were adhered to [20]. The number of interviews was determined by the level of saturation i.e. once no new information was being received, then no more interviews were conducted for each category of IDI [21]. The tapes were later transcribed and translated into English.

### Data management and analysis

The qualitative data from various sources were analyzed manually according to the themes of the study which included for the pre-school teachers; knowledge of STH signs, causes, transmission and prevention and awareness and for all sources; perceptions and experiences during NSBDP. The data was then triangulated for cross-verification to help increase the credibility and validity of the results by continuously cross-checking the data from the various sources. Quantitative data from the socio-demographic profiles was managed using excel spreadsheets.

## Results

### Background characteristics of the study participants

A total of 34 CHEWs, 41pre-school teachers, 38 primary school teachers, and 40 opinion leaders participated in the separate in-depth interviews. Table 1 presents the socio-demographic characteristics of all the study participants. The socio-demographic characteristics demonstrate the appropriateness of each category of participants for the study.

**Table 1:** Socio-demographic characteristics of the study participants in the IDIs

Description	Frequency (*N* = 154)	Percentage (%)
Gender		
Male	95	61.7
Female	59	38.3
Age in years		
20–24	8	5.2
25–29	21	13.6
30–34	25	16.2
35–39	15	9.7
40–44	20	12.9
45–49	24	15.6
≥ 50	41	26.6
Marital status		
Single	26	16.9
Married	126	81.8
Divorced	2	1.3
Religion		
Christianity	79	51.3
Islam	72	46.8
Missing	3	1.9
Occupation		
Chief/Assistant Chief	14	9.1
Business	5	3.2
Farmer	2	1.3
CHEW/PHO	34	22.1
Religious leader (Pastor or Imam)	5	3.2
School chairman	3	1.9
Village elder	9	5.8
Primary School Teacher	38	24.8
Pre-School Teacher	41	26.6
Youth leader	2	1.3

### Pre-school Teachers’ perception of soil-transmitted helminthes as a common problem among the Pre-school Age children

Of the 41 pre-school teachers interviewed, nearly all (*n* = 39) indicated that STH infections are a common problem among the PSAC and were referred to locally as *minyoo* or *minyolo*. However, on further probing, it emerged that about four-fifths (*n* = 32) of the respondents had a misperception that ringworms are in the category of STHs. Only about one-tenth (*n* = 4) were clear that ringworms are a skin fungal infection which is not due to worms and thus different from STHs. It was only one pre-school teacher who indicated that jigger infestation was more common than STHs.

A 28 year old female pre-school teacher from Malindi sub-county thus stated, “*Yes there is a possibility of having worms because even having ringworms it is a worm infection, roundworms and ringworm which are very common in the children*.”

### Pre-school Teachers’ knowledge of causes of soil-transmitted helminthes

With regards to awareness of causes of STHs, more than a half (*n* = 25) of the pre-school teachers had the knowledge of causes such as open defecation, poor hand washing practices and drinking untreated water. Not wearing shoes as a cause of STHs was mentioned by only about one-eighth (*n* = 5) and poor environmental sanitation by only about one-tenth (*n* = 4) of the study participants. Other causes of STHs mentioned included playing with soil (*n* = 12). A large majority of the pre-school teachers (*n* = 23) however had a perception that eating cold or uncooked foods was a cause of STHs.

A 38 year old female pre-school teacher from Malindi sub-county stated, “*Walking bare footed, eating dirty things can bring worms, I don’t remember the life cycle since it is long time since I was in school. The worms are spread when someone has worms, defecates and another person comes and steps on the feces they can get the worms.”*

A 48 year old female pre-school teacher from Matuga sub-county further stated, “*Most of the times for the children, they don’t wash their hands. They just eat, maybe they just pick a mango and start eating it, or get home, and just start eating their food… if they are hungry, they just eat it without washing hands. They just eat, dirty hands, dirty food. They are not keen on hygiene.”*

### Pre-school Teachers’ knowledge of signs of infection with STH

The common sign of worm infestation mentioned by the pre-school teachers was unhealthy looking hair and skin (*n* = 23). A small minority of the respondents indicated that irritation of the anus (*n* = 3) and blood in urine (*n* = 3) are signs of STH infection (Table 2).

**Table 2:** Pre-School Teachers’ perceived signs of worm infestation

Sign	Frequency of mention
Ringworms	32
Unhealthy hair and skin	23
Stomachache	14
Lack of appetite	12
Enlarged belly	12
Weak/malnourished body	9
Poor concentration in class/restlessness	8
Vomiting	8
Increased appetite	7
Diarrhea/frequent toilet visit	6
Blood in urine	3
Irritation in the anus	3

A 24 year old female pre-school teacher from Malindi sub-county stated that, “*Rashes, lack of appetite, loss of weight, unhealthy skin, and unhealthy hair. It is the worms that bring these symptoms.”*

A 35 year old female pre-school teacher from Msambweni sub-county further stated that, “*Children who are examined and found to have worms later get very, very sick and lose a lot of weight, such children keep on going to the toilet so we usually suspect they have that disease.”*

A 41 year female pre-school teacher from Matuga sub-county however stated, “*Like that one you see patches on the head and there are signs and symptoms that you see and those which you don’t see. Like the one that you see there are circles and there is no hair on the head so there you know the child has ring worms like that like that.”*

### Pre-school Teachers’ knowledge of transmission of STHs

With regards to the pre-school teachers’ knowledge of transmission of STHs, a large majority (*n* = 33) were aware that contaminated soil and poor water and sanitation practices are key modes. About one-fifth (*n* = 7) of the pre-school teachers however indicated that sharing of combs and soap are key modes of STH transmission (Table 3).

**Table 3:** Pre-School Teachers’ perceptions of causes of STHs transmission

Transmission	Frequency of mention
Open defecation	14
Walking barefoot/stepping on feaces	9
Lack of hand washing	10
Sharing of combs and soap	7
Eating unwashed fruits and vegetables	6
Drinking untreated water	6

A 41 year old female pre-school teacher from Matuga sub-county stated that, “*When you go to the bush, there are some who use bush, majority in this area they use the bush. May be last week someone else went there, and used that place and you when you pass you go to that same place you must step on that dirt.”*

A 44 year old female pre-school teacher from Malindi sub-county further stated that, “*Not washing hands, keeping long dirty nails, eating, kids playing and drinking dirty water and this makes the children have stomach pains, parents should take care of the children’s hygiene. I have forgotten about the life cycle and I think these causes are the ones that make the disease spread*.”

### Pre-school Teachers’ knowledge on prevention of STHs

On knowledge of how to prevent infestation with STHs, about four-fifths (*n* = 32) of the pre-school teachers mentioned deworming and health education. More than one-half (*n* = 23) indicated that observation of personal hygiene practices such as hand washing, toilet use and wearing shoes as well as environmental sanitation are measures for prevention of STHs.

The pre-school teachers were further asked to indicate whether they felt knowledgeable about STHs and nearly all (*n* = 40) reported that they required to have more knowledge on life cycle, causes, transmission and prevention. Slightly more than one-third (*n* = 14) of the pre-school teachers further indicated that they needed to learn about deworming for worm treatment.

A 38 year old female pre-school teacher from Matuga sub-county stated that: “*I have heard worms can be transmitted, may be you are in a house one of you has worms he can infect the other, now that is where I do not how he infects the other? Because if you treat one, the whole family must be treated. But now that transmission like that, me I don't know how it is transmitted until it gets to the other person*.”

### Pre-school Teachers’ experiences of community sensitization for NSBDP

Regarding how the pre-school teachers received information about deworming for pre-school age children, close to one-third (*n* = 12) reported that they were informed by the primary school teachers and about one- quarter (*n* = 10) by the health personnel. Only a minority (*n* = 5) indicated that they got the information from the radio or the village leaders (*n* = 4) or parents (*n* = 4) and education officers (*n* = 4). Two (2) of the pre-school teachers however indicated that they did not receive any information about the NSBDP. All the 41 pre-school teachers furthermore indicated that they needed to be involved in creating awareness to the community members about the deworming programme and would thus require first-hand information from the programme implementers.

A 38 years old female pre-school teacher from Malindi sub-county stated that: “*Yes, I was told by one of the village elders that the pre-school children needed to be treated so I went with the children to where the people were issuing drugs. I don’t remember the dates when the treatment was done, am thinking it is the month of March. It was done behind our nursery under the mango tree. They did not take long time to administer, they came after break time 10.30 am then by lunch time they were done.”*

### Pre-school Teachers’ experiences during deworming of Pre-school age children

More than a half (*n* = 23) of the pre-school teachers indicated that they assisted the CHEWs in some way during the deworming activity and that there were no problems experienced during and after the exercise although about one half (*n* = 20) indicated that some of the children were absent from school and therefore missed the treatment (Table 4).

**Table 4:** Pre-school Teachers’ mention of type of assistance they gave during deworming

How assisted?	Mention
Making the children queue and calling out their names	9
Treating those not willing to receive from the health worker	6
Washing hands	5
Record keeping	3

Furthermore a large majority (*n* = 26) of the CHEWs also indicated that the pre-school teachers were very supportive to them as they assisted in managing the children, helping them to wash their hands and encouraging them to chew and swallow the tablets as well as in record-keeping. However, of the 38 primary school teacher interviewed, less than one-quarter (*n* = 8) indicated that the pre-school teacher assisted them in managing the children, hand washing, encouraging them to chew and swallow the tablets and also in record-keeping.

A 38 years old female pre-school teacher from Malindi sub-county stated that: “*The kids were lined up and they were given each 1 tablet. I assisted the CHEW in dealing with the children who were afraid and did not want to take the drugs and because am not a stranger to them, so I gave them myself and they were able to take.*”

A 32 year old male CHEW from Malindi sub-county stated that: “*They assisted. They helped with writing of the names of the children in the forms and even giving the tablets and telling the children to queue and encouraging them to chew and swallow.”*

A 53 year old male CHEW from Msambweni sub-county further stated: “*In helping with the children, communicating with them, because you find that the children will confide more to the teachers and are freer. So, while lined up, when they are told that this is safe, at least there is that trust. So it was important for the pre-school teachers to be there.*”

Nonetheless about one-third (*n* = 14) of the pre-school teachers indicated that they were the ones who personally administered the drugs to the pre-school age children and that they did not encounter any problems. On further probing the pre-school teachers indicated that the CHEWs or the primary school teachers invited them for a session and instructed them on how to administer the treatment and left them to carry out the exercise. One pre-school teacher further indicated that she/he received the instructions through a telephone call from the CHEW.

Moreover less than one half (*n* = 16) of the primary school teachers interviewed indicated that they called the pre-school teachers and inducted them on how to go about drug administration, gave them the drugs and let them treat the pre-school age children. Two of the primary school teachers who allowed the pre-school teachers to administer the drugs indicated that they actually allowed them to carry the drugs and conduct the treatment at the pre-schools.

A 32 year old male primary school teacher from Lunga Lunga sub-county stated that: “*Since I was so busy treating my own pupils, as I was a class teacher at that time, I was not able to visit the pre-school teachers. But hoping they did just as I had trained them to, things were not that bad. To be frank I was not able to go about visiting them to observe. One of the madams asked me to visit them because she was a little stuck, but I directed her. And the pre-school teachers who didn’t know how to go about some areas came for consultation.”*

A 46 year old male primary school teacher from Msambweni sub-county further stated that: “*The pre-school teachers were of great help because there are some children who are below age four or five so when they have that normal teacher they become so happy they do not see that there is a strange face all together so they just took the drug.”*

### Pre-school Teachers’ perceptions of the NSBDP

On perception of the NSBDP, all the pre-school teachers indicated that the programme is very important and has helped in improving the health of the children. About one half (*n* = 20) indicated the children after being treated had healthier looking skin and hair and were less restless in class. Close to one half (*n* = 17) further indicated that more time should be allocated to deworming of the pre-school age children as the 1 day allocated was inadequate to attend to both the enrolled and the non-enrolled children and also because the pre-school age children are young and cannot be rushed in swallowing the drugs.

A 26 year old female pre-school teacher from Malindi sub-county further stated: “*It is a good thing; it helps the children that are being affected by worms. For example if a child has worms and the parents have refused to take them to hospital so when the drugs come it would be easy for the child to take and the child will become well. I just thank the programme, continue having that kind of a heart.”*

### Pre-school Teachers’ perceptions of method used to target the Pre-school Age children

Regarding the method used to target the pre-school age children, about three-quarters (*n* = 30) of the pre-school teachers indicated that the pre-school age children should be treated at their schools. The various reasons given for preference of treating the children at their schools included; the children are too young to be subjected to walk to the nearest primary school and parents not being happy with the children being made to walk and thus preferring them to miss school on the deworming day, the children being familiar with the pre-school teacher as opposed to a primary school teacher or a CHEW.

A 41 year old female pre-school teacher from Matuga sub-county stated that: “*Walking, walking will strain me because this road has big, big vehicles, those motor bikes, you may hold this the other one is jumping, jumping on the other side of the road, what if someone’s child is knocked? That is what I am telling you if I took children we went to the school there at maybe Viongwani Primary it will be difficult, the children are young imagine me taking the children there. That is why we were given those drugs we had to come and give them here ourselves*.”

Furthermore about two-thirds (*n* = 26) of the opinion leaders expressed the need to have the pre-school age children treated at their schools to avoid making them walk for long distances to the nearest primary school. The reasons cited for this preference included; parents not willing to have their children removed from their schools, insecurity of walking through bushes, having to cross rivers, causing congestion at the primary schools and subjecting them to strange environments at the primary schools.

A 56 year old male Assistant Chief from Malindi Sub-county thus stated: *“Children should be treated in the Pre-school, if they are told to walk these places have stones and forests and they are not able to walk. Last year they walked, and children got tired until parents said no it is not fair to our children. That was like punishing the little angels.”*

A 51 year old male CHEW in Malindi also stated *“The children should be treated at their Centres but the non- enrolled should be highly considered next time, the awareness and mobilization in the communities should be adequately done. As a CHEW I feel motivated to work with the Community.”*

### Pre-school Teachers’ perception of their capability to administer deworming treatment

All the pre-school teachers indicated that they had interest in administering the treatment to the children and would require to be trained by the programme implementers. Majority (*n* = 25) further expressed the need to have them involved in the deworming programme as this would help them acquire more factual information on worms and their control and thus impart proper life skills to the pre-school age children and play a role in encouraging the community members to adapt practices that would help control the infestation. The pre-school teachers further expressed the need to have them administer treatment to the pre-school age children citing this as an opportunity for them to learn more about worms deworming and feel motivated to assist in community sensitization for the programme.

A 40 year old female pre-school teacher from Msambweni sub-county further stated that: “*Mainly it is just that, you know now when me as the teacher I have known about worms I will be in a better position to educate those pupils and to also educate parents about worms. So if there is a way for me to know more about worms it would be good*.”

A 31 year old female pre-school teacher from Malindi sub-county also stated that: “*I need to be taught a lot that I don’t know. What I would like to know about worms is prevention ways and I want to be involved in educating others and also giving deworming drugs to the school children.”*

Moreover, about one-third (*n* = 14) of the opinion leaders supported the view that pre-school teachers could help in improving the programme by being empowered and getting involved in deworming of the children pointing out reasons such as; being more familiar and thus able to easily identify with the children, able to assist in community sensitization and in reaching the non-enrolled school age children. Other reasons given for need to empower the pre-school teachers were possibility of extending the deworming duration as more time was needed to administer drugs to the young children as it was not possible to rush them bearing in mind that their school programme lasts half a day.

A 47 year old male assistant chief from Msambweni sub-county stated that: *“Surely, if only you can incorporate the teachers of those schools to go and be empowered with the knowledge so that when they come back they have the message at hand with them. It looks very easy for them although they will be supervised by the health workers. I think that will reach very well to the learners because the teacher is dealing with the leaner himself or herself.”*

The primary school teachers further emphasized on the need to empower the pre-school teachers and have them administer treatment to the pre-school age children. Table 5 presents some reasons given by primary school teachers.

**Table 5:** Pre-school Teachers’ Perceptions of need to be empowered to conduct deworming activities

Reason for need to empower pre-school teachers	Mention
Children did not want to walk and were therefore absent	11
Mother school is far, 12 km away	7
Opted to walk there myself	7
Too many children to walk with one teacher	5
Did not get information therefore did not show up	3
Due to congestion left without being treated	3

## Discussion

The results reported in the current study indicate that pre-school teachers are aware that STH infections are common among the pre-school age children and are referred to locally as *minyoo* or *minyolo*. A repeated cross-sectional study conducted in Kwale County [22] reported overall baseline prevalence of between ≥20 and <50 % for any STH infection among school-age children and reported a latrine household ownership of 21.6 %. Results of a cross-sectional study conducted in the same county also reported high (41.7 %) hookworm prevalence among adult population and recommended community-wide treatment for control [23]. Evidently therefore STH infections are prevalent among all age groups living in the Region which can be attributed to poor practices of water and sanitation use.

Results of the current study demonstrate that majority of the pre-school teachers are aware that poor sanitation habits such as open defecation, poor hand washing practices and drinking untreated water are causes of STH infection. High level of awareness has been associated with decreased infection among children [24]. The teachers need to be empowered with practical skills and materials for use to create awareness among the pre-school age children and wider community. However, the current study results show that the pre-school teachers consider the consumption of cold food as a cause of STH infection. This is an indication of the need for awareness creation and health promotion campaigns to impart proper knowledge as has also been recommended by [25].

According to results of the current study there is awareness among the pre-school teachers about signs of STH infection such as unhealthy looking hair and skin, enlarged belly and loss of appetite. Similarly, a study on knowledge, attitudes and practices on schistosomiasis in Western Kenya reported high level of awareness of signs and symptoms [26]. However the study results also indicate that there are misconceptions among the pre-school teachers that ringworms are a sign of STH infection. Similar studies on awareness of signs of parasitic infections [27, 28] have also reported misconceptions on signs of infection. This demonstrates a knowledge gap that needs to be filled through health education which is an integral part of disease control.

The current study results also indicate that the pre-school teachers are aware that STH infections are transmitted through contaminated soil and unhygienic practices such as open defecation and lack of hand washing. The pre-school teachers should be encouraged to promote hygienic practices such as proper latrine use to prevent environmental contamination and hand washing among the pre-school children. However, Nyantekyi et al., reported low awareness of soil as a cause of infection and recommended implementation of preventive chemotherapy, health education and promotion of use of sanitary facilities to the community members to reduce morbidity and mortality due to STH [29]. Successful health promotion campaigns need to have interventions that put into consideration the behavior of the population being targeted similar to what was earlier recommended in another study [30]. Furthermore for consistent use of latrines in order to prevent environmental contamination, World Health Organization encourages provision of latrines that are in accordance with what already exists and is acceptable in the community [31].

The results reported in the current study also demonstrate that majority of the pre-school teachers were sensitized on the deworming programme through various modes such as by health workers and village leaders. The teachers however indicate that they are stakeholders who need to be involved in community sensitization which would require them to receive information from programme implementers and thus play a role of explaining the benefits of deworming and encouraging the parents to ensure that the children did not miss the treatment. Involvement of key players in health campaigns has been reported to contribute to motivation and thus increased compliance with treatment [32]. The current study results also indicate that the pre-school teachers played vital roles during the drug administration exercises and were key in the smooth flow of the process. Involving them fully can be regarded as a non-financial incentive that would encourage them to continue supporting the programme as reported in other studies [33, 34] and help in progressing towards achieving World Health Organization’s goal of increasing global PSAC deworming coverage to 75 % by 2020 [35].

The current study results show that the all pre-school teachers are aware of the benefits of the NSBDP on the health of the children and indicate a need to increase the treatment period so as to reach a large number of the children and also avoid rushing the young children in the swallowing of drugs. Similarly, results of a study conducted in Turkey [36] also reported 100 % awareness by school teachers of the importance of deworming children. The practical use and acceptability of schools as an entry point to deliver health intervention has been emphasized in a study conducted in the same study area in Kenya [37]. The school-based deworming programme implementers need to remember that a lot of patience is needed when handling the young children and consider extending the drug administration period for the pre-school age children and possibly invite other groups such as non-enrolled children and women of child bearing age. The system of having ‘treatment days’ as a promising strategy has been highlighted previously [38].

The results of the current study also demonstrate that the method used to reach the pre-school children is not considered effective by the pre-school teachers, primary school teachers as well as opinion leaders who consider it a punishment and a security risk to have the children walk to the nearby primary schools in view of the fact that some are as far as 12 km away. There is also a feeling of confidence among the study participants that the pre-school teachers can conduct the drug administration especially because the young children are familiar with them further endorsing the need to involve them in the worm control campaign.

The current study results have also demonstrated that the pre-school teachers have interest in participating in the deworming programme and being empowered would help them play integral roles such as imparting knowledge on water and sanitation use to the children. The pre-school teachers would also be useful in sensitizing the community on the benefits of deworming thus increasing compliance with treatment since the children would not be absent fearing being expected to walk to primary schools. The World Health Organization [39] has attributed success of school-based deworming programmes to be related to the community’s acceptability of the teacher’s role.

## Conclusion

The findings of this study have shown that pre-school teachers are important contributors to the existing School-based deworming programme. They need to be empowered with adequate information on STH and School-Based deworming. Extension of the treatment duration for the pre-school age children should be a good strategy of increasing compliance and coverage among other high risk groups during the campaign. With proper information, the teachers can instill knowledge on water and sanitation use to the young children and also play a role in community mobilization for NSBDP. County Governments who are the current employers of the pre-teachers need to find ways of utilizing them in similar health programmes.

## Limitations of the study

This was purely a qualitative study and the purpose was to explore and describe knowledge and perceptions of soil-transmitted helminthes infection and control among pre-school teachers as well as assess their experiences during NSBDP. All the study participants were purposively selected. The results are not generalizable as the sample size of a qualitative study is not representative but can be used to build theory through tentative hypotheses. The results will inform the NSBDP management on considerations to make during future planning.

## Abbreviations

CHEWs: Community Health Extension Workers
IDIs: In-Depth Interviews
KEMRI: Kenya Medical Research Institute
MCH: Maternal child health
MDA: Mass Drug Administration
NACOSTI: National Commission for Science and Technology and Innovation
NSBDP: National School-Based Deworming Programme
NTDs: Neglected tropical diseases
PSAC: Pre-school age children
SAC: School-age children
STH: Soil-transmitted helminthes
STI: Science Technology and Innovation
WHO: World Health Organization
